# Diffuse large B cell lymphoma in a preceding IgG4-related disease with kidney restricted lambda light chain expression: case report and literature review

**DOI:** 10.1186/s12882-020-01975-7

**Published:** 2020-07-29

**Authors:** Hui Wang, Tao Su, Lei Kang, Li Yang, Suxia Wang

**Affiliations:** 1 Renal division, Department of medicine, Peking University First Hospital, Peking University Institute of Nephrology, Beijing, China; 2 Renal Pathology Center, Peking University First Hospital, Peking University Institute of Nephrology, Beijing, China; 3 Laboratory of Electron Microscopy, Pathological Center, Peking University First Hospital, Beijing, China; 4 Department of Nuclear Medicine, Peking University First Hospital, Beijing, China

**Keywords:** Diffuse large B cell lymphoma, IgG4-related disease, Restricted light chain expression, Extranodal marginal zone lymphoma, Case report

## Abstract

**Background:**

IgG4-related disease (IgG4-RD) is a newly classified but poorly understood immune-medicated systemic disease. It causes potential fibroinflammation in one or more organs, characterized by tumescent organs and marked IgG4-positive plasma cells infiltration in the affected tissues. There have been a few cases revealing close relationship between IgG4-RD and formation of B cell lymphoma. Diffuse large B cell lymphoma (DLBCL) and extranodal marginal zone lymphoma (EMZL) of mucosa-associated lymphoid tissue are the most common sub-types ever described, whereas the exact mechanism remain unclear.

**Case presentation:**

We report a 64-year old Chinese male who presented chronic kidney disease and was initially diagnosed typical IgG4-RD. Pathological findings revealed there was restricted expression of lambda light chain in the kidney. There was also elevated uptake abnormality observed in ^18^F-FDG-PET/CT. Prednisone combined with oral cyclophosphamide helped the patient to get a partial remission of renal function and an obvious decrease of IgG4 level. However, he developed DLBCL 16 months after IgG4-RD diagnosis. The DLBCL is speculated to transform from a pre-existing but possible missed diagnosed EMZL.

**Conclusions:**

Concurrent IgG4-RD with kidney-origin EMZL developing DLBCL has never been reported in the literature. Clinicians should keep in mind that lymphoma may occur in IgG4-RD. The mechanism of lymphomagenesis potential in IgG4-RD needs further study.

## Background

IgG4-related disease (IgG4-RD) is a newly classified but poorly understood immune-medicated systemic disease. It causes potential fibroinflammation in one or more organs, characterized by tumescent organs and marked IgG4-positive plasma cells infiltration in the affected tissues. This chronic inflammatory state is thought to be associated with a high incidence of lymphoma during the course of the disease. There have been a few case reports revealing concurrence of IgG4-RD and B cell lymphoma. But the exact pathogenesis remain unclear.

We describe a 64-year old Chinese male who presented chronic kidney disease and was firstly diagnosed typical IgG4-RD and IgG4-related tubulointerstitial nephritis. There was also restricted expression of lambda light chain in the kidney and extremely elevated uptake observed in ^18^F-FDG-PET/CT. The patient developed DLBCL 16 months after IgG4-RD diagnosis. The DLBCL is speculated to transform from a pre-existing but possible missed diagnosed EMZL.

## Case presentation

A 64-year old Chinese male was admitted to hospital for investigation of renal dysfunction. He was asymptomatic when his serum creatinine was found to be 143.9 μmol/L in routine medical examination 1 year ago. Subsequent laboratory testing showed marked hypergammaglobulinema along with slight hypocomplementemia (IgG 46.9 g/L, C3 0.59 g/L, C4 0.06 g/L). The patient complained loss of appetite for the recent 2 months before hospital admission. As a retired sewing worker and also a heavy smoker, he was diagnosed interstitial lung disease by CT for 6 years.

**Fig. 1:**
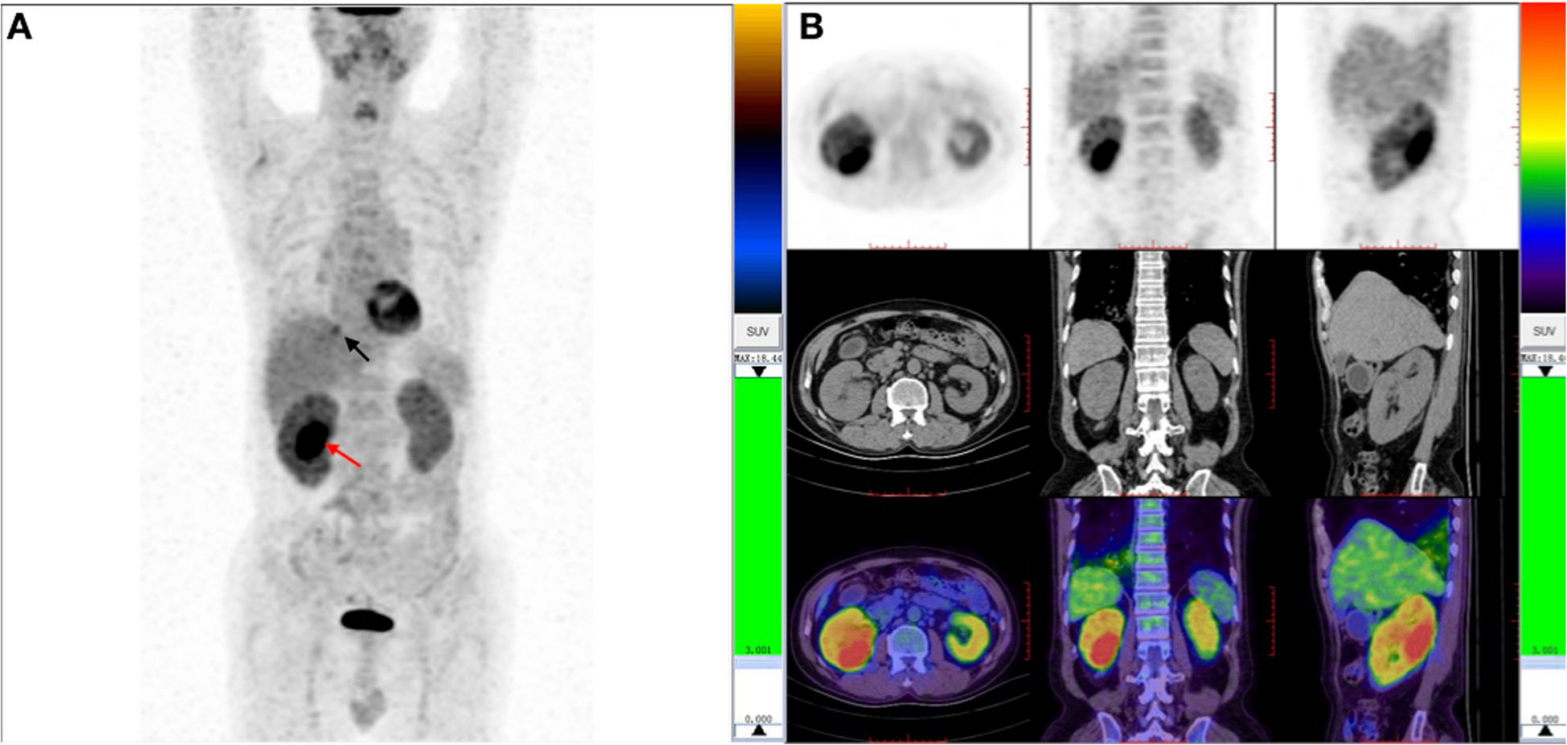
18F-FDG PET / CT images: There were bilateral plump kidneys and FDG slightly avid (SUVmax 4.6). There was no obvious abnormal density change in the local mass of the right dorsal kidney, but FDG uptake was significantly increased (SUVmax 11.1) and the concentration range was about 28 mm × 42 mm × 50 mm (AP × RL × SI) (red arrow). The interlobular space of the lower lobe of both lungs were thickened and changed in grid-like manner, with the posterior basal segment and the outer basal segment of the right lower lobe as the most obviously. The FDG uptake increased, and SUVmax was 5.1 (black arrow)

On admission, laboratory testing showed serum creatinine level was 196 μ mol/L, erythrocyte sedimentation rate was 24 mm/h, the albumin level was 31.3 g/L in the presence of mild proteinuria (0.29 g/day) with small molecular weight proteins accounting for 62.1% of the total. There was a polyclonal hypergammaglobulinemia (IgG1 16.6 g/L, reference 4.9–11.4 g/L; IgG2 4.58 g/L, reference 1.69–7.86 g/L; IgG3 2.4 g/L, reference 0.11–0.86 g/L; IgG4 34 g/L, reference 0.03–2.01 g/L) because M-protein was negative both in serum and urine. The level of complement C3 was obviously decreased to 0.405 g/L, C4 was 0.031 g/L. The antinuclear antibody was negative. Doppler ultrasonography and abdominal CT revealed enlarged kidneys. There was no evidence of masses in kidneys and other organs or signs of retroperitoneal fibrosis in CT image. ^18^F-FDG-PET/CT scan showed diffuse high uptakes in bilateral submaxillary glands (SUVmax 4.3), kidneys (SUVmax 4.6) and interlobular septa (SUVmax 5.1). There was extremely elevated uptake in a local region of the right kidney (28x42x50 mm, APxRLxSI), the SUVmax was 11.1 (Fig. 1). No enlarged lymph node was detected by ultrasound and ^18^F-FDG-PET/CT.

**Fig. 2:**
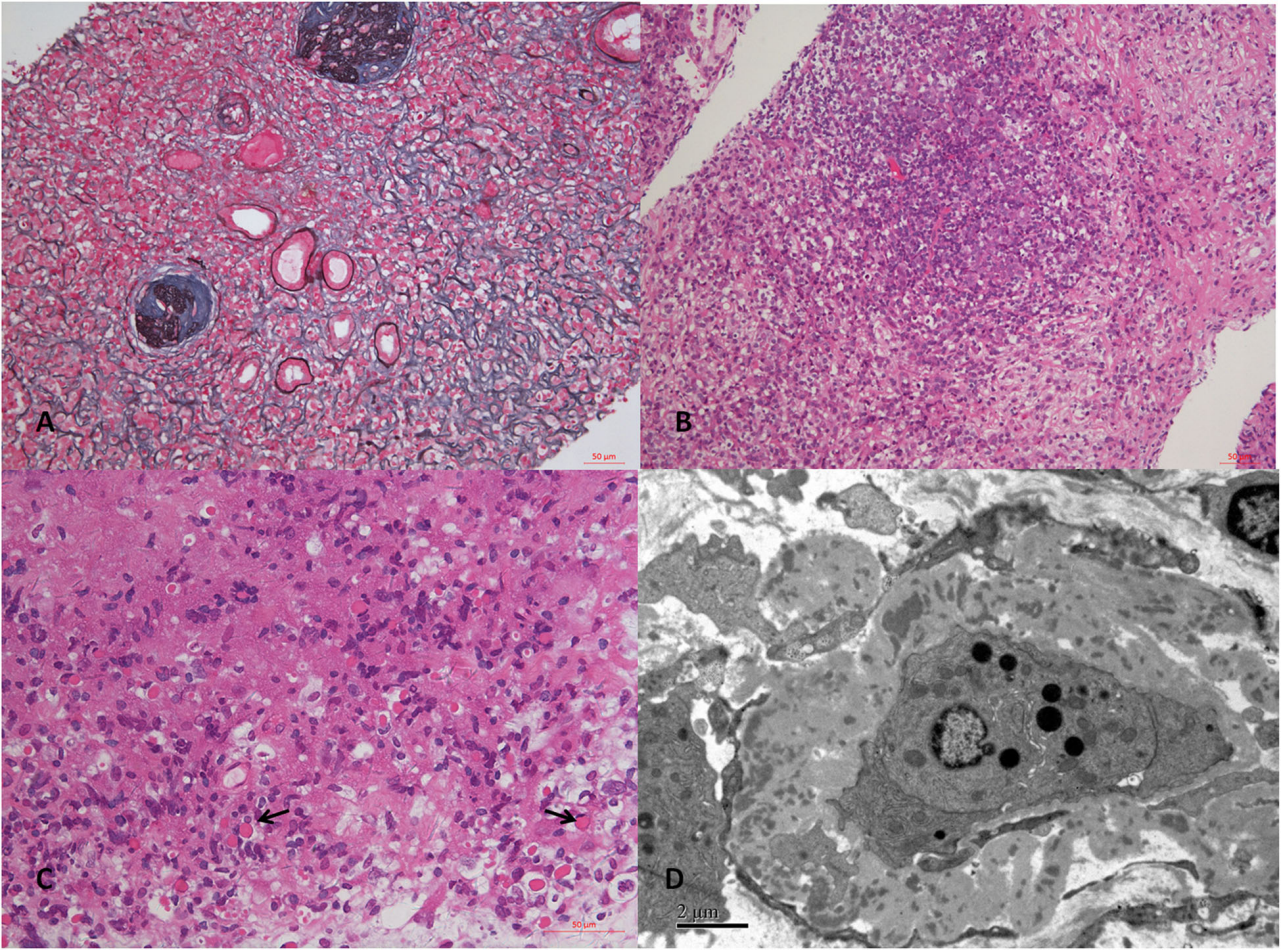
Representative light microscopic findings of renal biopsy at first diagnosis. **a**. Marked “storiform” fibrosis was seen in the interstitium. The glomeruli showed ischemic sclerosis (Masson trichrome-methenamine silver, × 200). **b**. Ectopic germinal center-like structure was formed in the interstitium (HE, × 200). **c**. Russell body and a few “diamond” or “rectangle” crytals can be seen in some plasma cells (HE, × 400). **d**. Electron dense deposits were detected along tubular basement membrane (TBM) (× 6000)

**Fig. 3:**
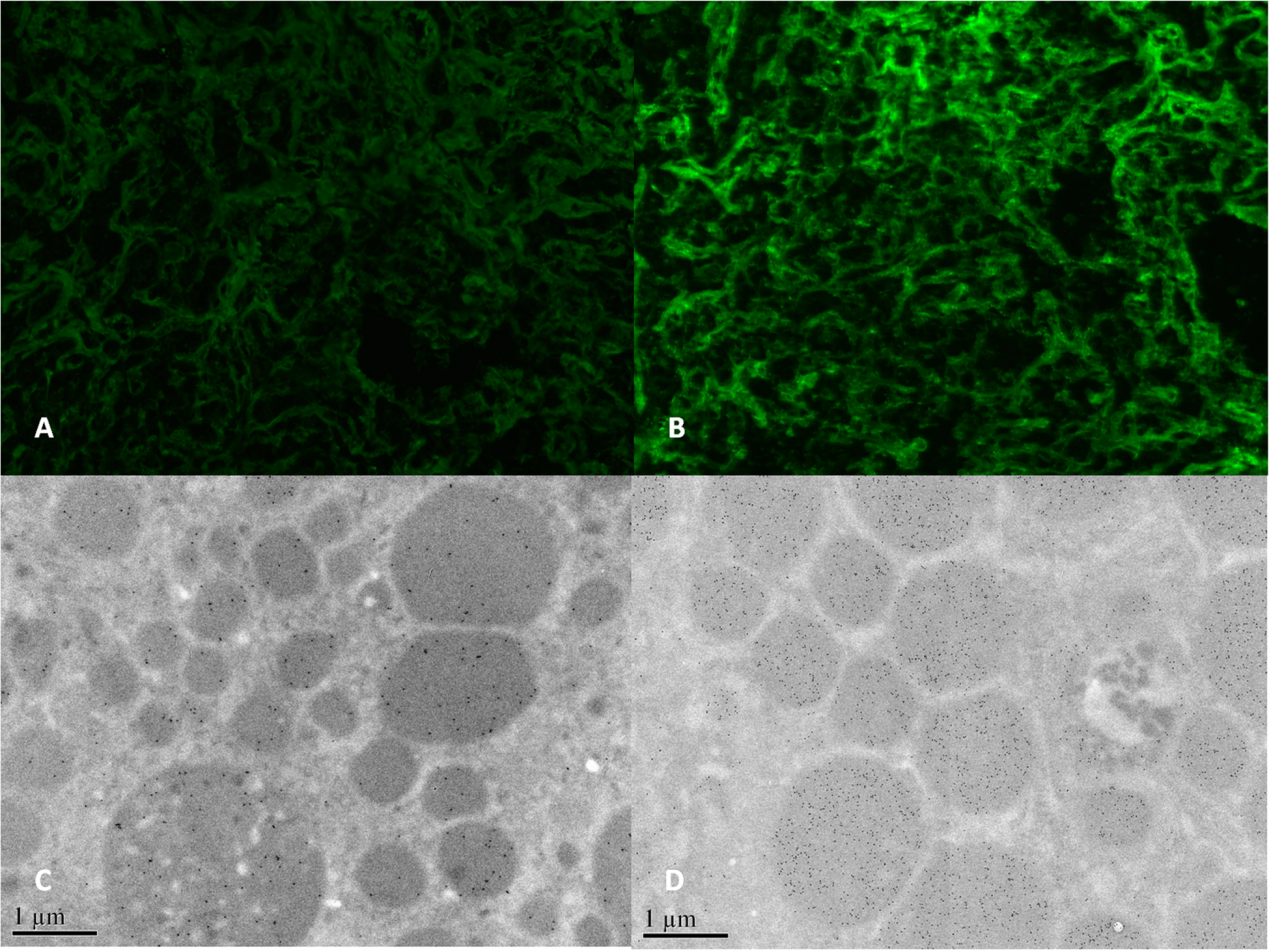
Representative immunohistochemistry findings of renal biopsy at first diagnosis. **a**. Mild staining of immunoglobulin lightκin the interstitium (Immunofluorescence staining,× 200). **b**. Moderate to strong staining of immunoglobulin lightλin the interstitium (Immunofluorescence staining,× 200). **c**. Scattered immunoglobulin lightκwere detected in the plasmacytoid cells. (Immunoelectron microscopy labelling, × 25,000). **d**. Intense immunoglobulin lightλwere detected in the plasmacytoid cells. (Immunoelectron microscopy labelling, × 25,000)

The patient underwent routine ultrasonic guided right kidney biopsy. By light microscopy, there were 16 glomeruli in biopsied samples, 9 glomeruli were ischemic sclerosis. Pathological findings revealed a diffuse lymphoplasmacytic interstitial infiltrate that included lymphocytes, macrophages, plasma cells and eosinophils. The hallmark storiform fibrosis could be seen (Fig. 2a). Electron dense deposits were detected along tubular basement membrane (TBM) (Fig. 2d). IgG4 positive plasma cells count more than 10 cells per high power field. And the ratio of IgG4 to IgG positive cells was 50%. These findings conformed to a diagnosis of IgG4-related tubulointerstitial nephritis. In addition, the formation of ectopic germinal center-like structure was detected in focal interstitium, which was characterized by the clustering of T: B cells, with plasma cells scattered around the clustering area, similar as lymphofollicular-like structure (Fig. 2b). Russell body and a few “diamond” or “rectangle” crytals can be seen in some plasma cells (Fig. 2c). Notably, the immunofluorescence staining showed there was abundant deposition of immunoglobulin light chain (λ) in the interstitium compared with κ (Fig. 3a and b). Furthermore, there was also much more expression of immunoglobulin λ light chain in the plasmacytoid cells compared with κ by electron microscopy immuno-gold labelling (Fig. 3c and d). However, there was no evident monomorphic cell proliferation. Congo red staining was negative. Taken together, the high uptake disclosed in FDG-PET/CT image was consistent with the pathological findings indicating there were typical IgG4-RD and restricted de novo light chain expression.

**Fig. 4:**
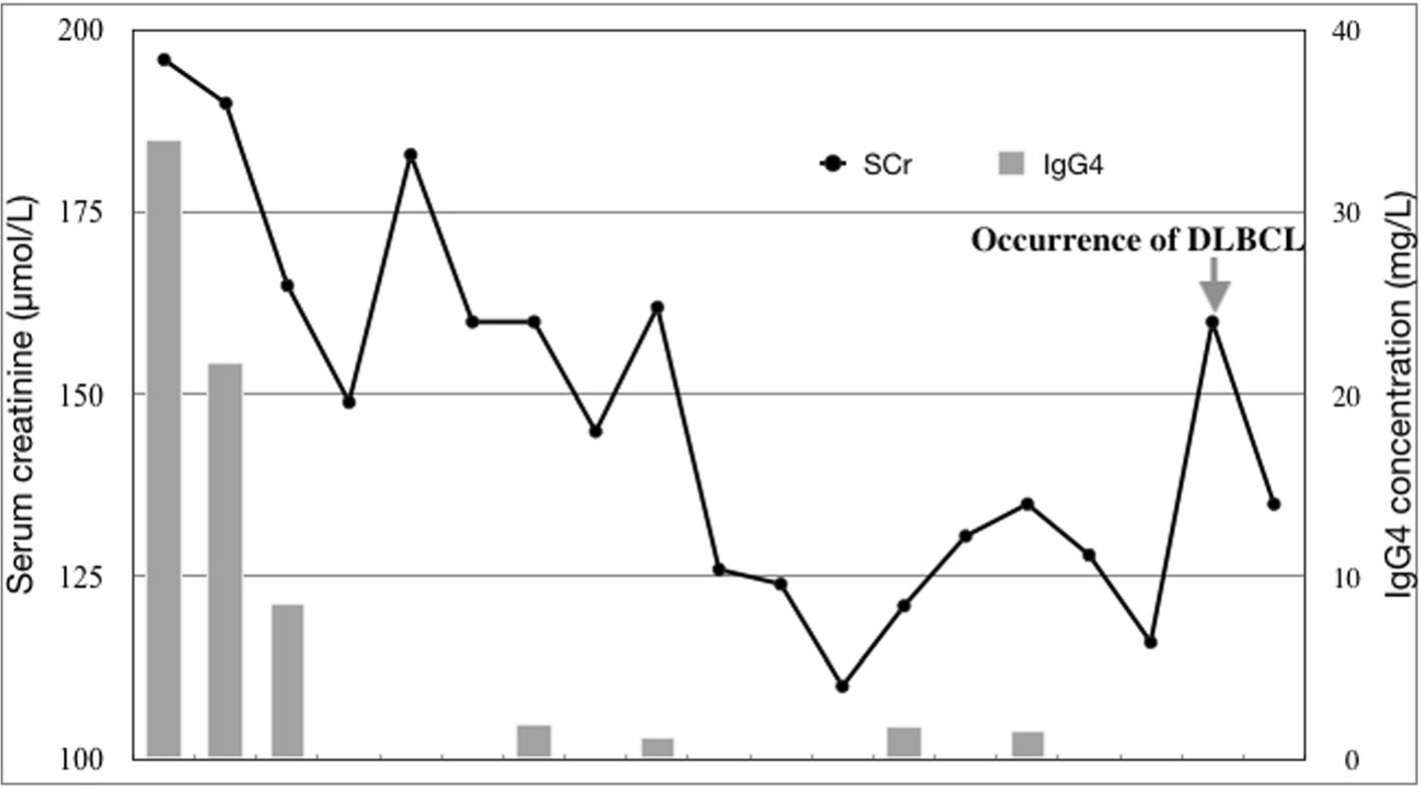
Changes of SCr and IgG4 level during follow-up

The patient was mobilized for bone marrow biopsy, but unfortunately the patient refused at that time. He was finally diagnosed IgG4-RD, multiple organs involved of kidneys, bilateral submaxillary glands and interlobular septa. He was prescribed oral prednisolone at an initiated dose of 40 mg/day for 6 weeks then tapered. Symptoms relieved soon and serum creatinine declined gradually to 149 μmol/L at the 12-week after prednisolone initiation. With combination of oral cyclophosphamide (50 mg/day started from the 7th week for the following 20 weeks, the cumulative dose was 7 g), the IgG4 concentration was reduced to 1.27 g/L and maintained at 1.56 g/L with a daily dose of prednisolone 5 mg (Fig. 4). Unfortunately, the patient developed a sudden enlargement of left cervical lymph nodes with fever 16 months after IgG4-RD diagnosis. Histopathological assessment of the biopsied cervical lymph node identified diffuse infiltration of abnormal large lymphoid cells that were positive for CD20 and MUM-1, but negative for CD3, CD5, CD10, BCL-6. CD38 was strongly positive in the cytoplasm of some neoplastic cells (about 40%). The Ki-67 index was 70%. In situ hybridization for Epstein-Barr virus-encoded RNA (EBER-ISH) was negative. These findings conformed to a diagnosis of DLBCL, non-GCB subtype (Hans algorithm). Malignant cells stained positively for MYC (about 40%) and BCL2 (about 80%). However, fluorescence in situ hybridization (FISH) suggested the absence of MYC, BCL2 and BCL-6 rearrangements. ^18^F-FDG-PET/CT showed remarkably high uptake of multiple lymph nodes in cervical, supraclavicular, submaxillary and peri-abdominal aorta areas. The SUVmax value was 15.1–22.3. However, The previous high uptakes of kidneys had completed disappeared. The patient received periodical chemotherapy of R-DA-EPOCH strategy. His serum creatinine fluctuated within a short time and eventually was stable at 125 μmol/L in a recent follow-up.

## Discussion and conclusions

IgG4-related disease (IgG4-RD) is a newly classified but poorly understood immune-medicated systemic disease. It causes potential fibroinflammation in one or more organs, characterized by tumescent organs and marked IgG4-positive plasma cells infiltration in the affected tissues, often with elevated serum IgG4 level [1]. The IgG4-RD is diagnosed basing on clinical and pathological presentations according to the 2011 IgG4-RD diagnostic criteria. The mechanisms of underlying immune abnormalities to IgG4-RD remain unclear. There have been a few cases revealing close relationship between IgG4-RD and formation of B cell lymphoma [2], the most common type was non-Hodgkin lymphoma (NHL). It is reported that the overall incidence of NHL was increased in patients with IgG4-RD when compared to general population (standardized incidence ratios 400.00 [95%CI 109.00–1024.00] [2]. Although this was a retrospective observational study with a relatively short follow-up period, an etiologic link between IgG4-RD and lymphoma deserved more attention. As listed in Table 1, several research groups have reported single cases or small case series developing malignant lymphoma with a preceding IgG4-RD [3–15]. Up to date, the number of published cases is only 25. DLBCL and EMZL of mucosa-associated lymphoid tissue are the most common sub-types ever described in the literature. The special case we present here is a patient developing non-GCB type DLBCL 16 months later after his diagnosis of IgG4-RD. The preceding IgG4-RD was typical with lambda light chain locally restricted expression, suggesting a possibly missed diagnosis of EMZL in the right kidney. As we all know, the diagnosis of EMZL can be rather challenging, as extranodal sites of disease are sometimes difficult to access, resulting in the limitation of small biopsy samples. The optimal diagnosis of EMZL requires integration of clinical, histopathological, and molecular information [16].

**Table 1:** Clinical profiles of recently reported cases of IgG4-related disease with malignant lymphoma

Case No.	Author	Age range	Primary IgG4-RD	Type of malignant lymphoma	Interval to lymphoma (years)
1	Cheuk W	60–70	Chronic Sclerosing Dacryoadenitis	FL	3
2	Cheuk W	60–70	Chronic Sclerosing Dacryoadenitis	EMZL	1
3	Cheuk W	60–70	Chronic Sclerosing Dacryoadenitis	EMZL	1
4	Takahashi N	60–70	Autoimmune pancreatitis	B cell lymphoma	4
5	Takahashi N	60–70	Autoimmune pancreatitis	DLBCL	5
6	Takahashi N	60–70	Chronic parotitis	DLBCL	3
7	Kanda G	60–70	IgG4-related sclerosing cholangitis	PTCL	2
8	Yamamoto M	30–40	dacryoadenitis	EMZL	1
9	Mitsuyama T	70–80	IgG4-related prostatitis	DLBCL	3
10	Ishida M	60–70	Autoimmune pancreatitis, IgG4-related cholecystitis	DLBCL	5
11	Kase S	60–70	IgG4-related inflammation of the orbit	EMZL	3
12	Lightfoot N	60–70	IgG4-related pachymeningitis	DLBCL	concurrent
13	Mulay K	60–70	Dacryoadenitis	EMZL	concurrent
14	Nishimura Y	60–70	Autoimmune pancreatitis	DLBCL	4
15	Kawaji Y	60–70	IgG4-related lymphadenopathy	DLBCL	4
16	Xiaolin Peng	40–50	IgG4-related ophthalmic disease	DLBCL	concurrent
17–25	Bledsoe JR	22–68	IgG4-related disease	DLBCL:4 EMZL: 2 FL:1 Lymphoplas-macytic lymphoma:1	Concurrent:2 Asynchro-nous: 4.3–16.4

*FL* follicular lymphoma, *EMZL* extranodal marginal zone lymphoma, *DLBCL* diffuse large B-cell lymphoma, *PTCL* peripheral T-cell lymphoma

The underlying pathophysiologic mechanisms that may potentially contribute to lymphomagenesis in IgG4-RD are poorly defined. Chronic inflammation is a known predisposing factor for increased risk of malignant lymphoma including DLBCL. It could establish an environment fertile to lymphoma development in both nodal and extranodal sites especially EMZL [17, 18]. The chronic inflammation might be induced by bacteria, virus or various autoimmune diseases including IgG4-RD [8, 16–23]. We have known that there is fibroinflammatory condition in IgG4-RD. Data suggested that the disease-associated oligoclonal plasmablasts expansions and the T-dependent B-cell activation events contribute to the persistent immune inflammation, represent body responses to self-antigens, and likely drive IgG4-RD disease progression. Plasmablasts are defined as CD19+ CD20- CD38+ bright CD27+ on the CD19+ lymphocytes population gate. They are the precursors of tissue resident antibody secreting plasma cells with oligoclonal and exhibit extensive somatic hypermutation. The number of plasmablasts is an indicator of IgG4-RD disease activity. It is reported that de novo oligoclonal expansions of circulating plasmablasts change along with activation and relapse of IgG4-RD, might be responsible for the chronic inflammation [24, 25]. Plasmablasts further differentiate and proliferate in peripheral lymph tissue to form mature plasma cells and produces antibodies. Some pathologists have noticed the structure of the lymph node germinal center appeared in the affected organs of IgG4-RD. Indeed, our previous study also showed ectopic lymphoid like structures located in 66.7% kidneys with IgG4-related tubulointerstitial nephritis, and increased Russell body formation in renal interstitial plasma cells [26]. These are potential explanations for the abundant lymphocytes and plasma cells in the interstitium and antibodies production. Chronic inflammation under immune stimuli leads to local aggregation and proliferation of antigen-dependent B and T cells.

In the present case, DLBCL developed 16 months after the first diagnosis of IgG4-RD. The occurrence of DLBCL is related either to the development of DLBCL de novo or to the transformation from EMZL. According to the literature, the rate of histological transformation to a DLBCL has been reported to be in the range of 2–5% for EMZL, with the median time for transformation being 11–48 months [27–30]. Interestingly, the large majority of DLBCL following EMZL is clonally-related, which constitutes a real transformation between EMZL and DLBCL. Moreover, a study from Russia further confirmed clonal relationship of EMZL and DLBCL in Sjogren’s syndrome patients, which most likely shows that high-grade DLBCL emerged from low-grade EMZL in Sjogren’s syndrome patients [31]. In our case, while EMZL possibly existed at the initial diagnosis on the background of IgG4-related disease, transformation to aggressive B-cell lymphoma may occur. DLBCL has been stratified by gene expression profiling into two major groups associated with their cells of origin: the GCB subtype with a better prognosis and the non-GCB subtype with a worse prognosis [32]. DLBCL that occurs in various autoimmune diseases, such as systemic lupus erythematosus and Sjögren syndrome, is mainly related to the non-GCB subtype of DLBCL [31, 33]. In the present case, the DLBCL showed non-GCB subtype and well CD38 expression, which was consistent with the pathogenesis hypothesis of chronic B-cell stimulation and antigenic drive.

In summary, this case was confirmed to have a IgG4-RD with kidney involvement as tubulointerstitial nephritis. It is noteworthy that this case also showed lambda light chain restriction, indicating the probable existence of oligoclonal expansion of IgG4 positive circulating plasmablasts at initiation. An EMZL might exist on the background of IgG4-related disease, and histological transformation to aggressive B-cell lymphoma may be possible. Concurrent IgG4-RD with kidney-origin EMZL developing DLBCL has never been reported in the literature. This case further expanded the pool of potential sites of tumourigenesis in the entity of IgG4-RD. Most important, clinicians should keep in mind that lymphoma may occur in IgG4-RD. Researches focusing on disease pathogenesis and malignant potential are necessary in the future.

## Data Availability

The patient was regularly followed up and the clinical data is traceable. The datasets used and analysed during the current study are available from the corresponding author on reasonable request.

## Abbreviations

IgG4-RD: IgG4-related disease
DLBCL: Diffuse large B cell lymphoma
EMZL: Extranodal marginal zone lymphoma
NHL: Non-Hodgkin lymphoma
